# The Invisible Patient: Concerns about Donor Exploitation in Stem Cell Research

**DOI:** 10.1007/s10728-022-00448-2

**Published:** 2022-11-25

**Authors:** Pär Segerdahl

**Affiliations:** Centre for Research Ethics and Bioethics, Department of Public Health and Caring Sciences, Uppsala University, Box 564, SE-751 22 Uppsala, Sweden

**Keywords:** Egg donation, Embryonic stem cell research, Exploitation, Thick ethical concepts, Gift relationship

## Abstract

As embryonic stem cell research is commercialized, the stem cell debate may shift focus from concerns about embryo destruction to concerns about exploitation of the women who donate eggs and embryos for research. Uncomfortable with the polarization of the embryo debate, this paper proposes a more “contemplative” approach than intellectual debate to concerns about exploitation. After examining pitfalls of rigid intellectual positions on exploitation, the paper investigates the possibility of a broader understanding of donation for research where patients are seen as the intended beneficiaries of the donation. Together with other actors, research is perceived as mediating altruistic gift relationships that extend from donors to patients. The paper explores how this broader perspective on “donation for research” can open up new possibilities of understanding donation and addressing risks of exploitation.

## Introduction

Debates about the ethical permissibility of embryonic stem cell research almost exclusively focus on the moral status of the embryo. However, the research also has another sensitive aspect. Since it relies on a supply of fresh ova and frozen embryos, and since commercial interests increasingly are interwoven with the research, worries about exploitation need to be seriously considered. But are they? In an article entitled, “The lady vanishes,” Donna L. Dickenson [1] called attention to a missing debate, addressing the role of women in the stem cell technologies:

In most public discussion of the ethical issues in stem cell research, only the status of the embryo seems to count. Yet because ova are crucial to stem cell research, there are also important regulatory issues concerning protection of women from whom ova are taken … In most cases and debates, the women from whom the ova are taken have virtually disappeared from view. [1: 43]

More recently, Søren Holm noticed how “arguments relating to the interests of embryo and gamete donors are curiously absent from the particular stem cell banking policy discourse” [2: 265]. He also issued a warning to the field of embryonic stem cell work:

Although some in the stem cell field see themselves as outside of the sphere of reproduction and reproductive policy, it is not obvious that society sees it that way. A lack of proper attention to the rights and interests of embryo and gamete donors in relation to stem cell derivation may over time undermine policy support for the field. [2: 275]

One could wonder why donor interests and risks of exploitation do not figure prominently in policy discussions on embryonic stem cell research. It may, I surmise, partly be due to a fear that concerns about donor exploitation could trigger an equally polarized debate as the concerns about embryo destruction did. With the debate about whether embryo destruction is murder in fresh memory, some in the stem cell field may fear a second polarized debate, this time about whether women are exploited in the stem cell industries. If Holm is right, however, sweeping such concerns under the rug may in the long run prove counterproductive. And unethical, one could add.

In this paper, I suggest that a second polarized stem cell debate is not improbable; that concerns about exploitation of donors need to be voiced rather than silenced; and finally, that bioethics needs to approach these concerns more cautiously and “contemplatively” than in debates for and against doctrines. A contemplative approach to an issue as serious as exploitation can itself seem provocative, of course, because the openness of such an approach could seem to imply openness to exploitation. However, that is why patience and caution are needed. We are considering sensitive normative notions like murder and exploitation, which can be contested in their application to embryonic stem cell research. We need to be aware of how these concepts provide quite worrying images of research practices, and that this property of the images risks preventing an open discussion about the applicability of the concepts. The discussion in this paper is therefore not based on any specific definition of exploitation and will not propose one, but focuses on this general difficulty in concepts that have both descriptive and evaluative aspects. Later in the paper, an attempt in the literature to define exploitation to fit egg donation for research will be examined from this point of view.

## Intellectual Dangers of Thick Ethical Concepts

Bernard Williams [3] distinguished between thick ethical concepts such as “brave” and “brutal,” which have both descriptive and evaluative aspects, and thin ethical concepts such as “right” and “wrong,” which are purely evaluative and action-guiding. “Murder” and “exploitation” can be understood as thick ethical concepts. They have a descriptive aspect combined with a strong negative evaluative action-guiding aspect.

Although the two aspects cannot be separated, this duality of thick ethical concepts, their descriptive-normative Janus face, makes them useful for ideological purposes. If you oppose X, and can demonstrate that X, *in fact*, involves murder or exploitation (descriptive aspect), then you immediately seem to have demonstrated that X *must* be condemned (normative aspect). Thick ethical concepts have been used in conflicts to legitimize actions against people who were described as unreliable, greedy, exploitative and even murderous. Since the words are assumed to describe reality, the applicability of the concepts seems to justify us to both condemn and take action against these people.

In relation to the use of thick ethical concepts for ideological purposes, I want to mention three common intellectual dangers or temptations of such concepts.

Dogmatism: The first is that it can be difficult to raise questions about the applicability of such concepts, since it might seem as if you questioned their evaluative aspect. Let us say that you raise the question if embryo destruction is really murder. In the eyes of those who take this description for reality, you can appear like someone who does not take the negative evaluative aspect of the concept seriously. Just asking the question may seem suspicious. The very openness of the question already seems to speak against it and can evoke reactions such as: “Murder is *not* something to be open-minded about!”

Righteousness: A second troublesome feature is that thick ethical concepts easily produce a good self-image for any ideological movement. Any ideology is on the right side, regardless of which side it is on, since it strives for what its moral vocabulary unites with the good, and opposes what its vocabulary unites with the bad. Any ideology seems to have right and duty to act against what its thick moral vocabulary picks out as blameworthy features of reality.

Moral anxiety: A third problem is that thick ethical concepts can produce anxiety in the form of gnawing suspicions and fears. Most of us are not familiar with embryonic stem cell research, we do not know for sure what it is. Thick ethical concepts can then act as a substitute for what we do not know. They appear in the form of an inner voice that tells us what stem cell research *is*. This is not a purely descriptive “*is*,” but a double-edged one, for what the voice in the head says the research *is* could be a frightening, “It *is* murder.” Since we are ignorant of much, but not of our anxiety, we cannot shake off the thick ethical concepts that have begun to spin in our heads. They seem validated by the anxiety they produce, which is real, and we worry endlessly, caught in a whirlpool of thick descriptive-normative moral language and fear.

In pointing out these dangers of thick ethical concepts, I am not questioning their important functions in our language. It is difficult to imagine human life without these concepts. I am just pointing out how the dual nature of thick ethical concepts can sometimes lock our perspective on reality and make debates on important issues uncompromising. I think many of us have experienced getting caught up in such “thick” descriptions of reality.

Precisely because concerns about donor exploitation deserve careful attention, we need to be aware of the intellectual dangers of thick ethical concepts. The word “exploitation” easily puts us in a state of emergency and can trigger reactions as if we were facing an imminent threat. And that is our difficulty. The investigation is motivated by a concept that simultaneously threatens to short-circuit the investigation.

## A Second stem cell war?

Although Dickenson [1] makes very important observations on a missing issue in the stem cell debate, she does not seem to particularly emphasize the problems surrounding its sometimes almost war-like nature. On the contrary, the metaphor is used to emphasize the importance of another front line:

What unites the two warring sides in “the stem cell wars” is that women are equally invisible in both: “the lady vanishes.” Yet the most legitimate property in the body is that which women possess in their reproductive tissue and the products of their reproductive labour. [1: 43]

I agree, of course, that concerns about women’s status as donors are at least as important as concerns about the status of the embryo and should be highlighted in the discussion about stem cell research. The question I want to raise in this paper is whether these concerns are best addressed by what could be described as a second stem cell war, where the spirit might sometimes be one of using “whatever weapons are available to us” [1: 53]?

Already the first debate, about the embryo, polarized debaters and exhibited tendencies towards dogmatism, righteousness, and moral anxiety [4]. My question is therefore whether the discussion of concerns about donor exploitation risks becoming a second stem cell war that reproduces similar problematic tendencies. Consider this way of initiating debate about exploitation in stem cell research, by Heather Widdows:

This article will argue that as practices *qua* practices, both trafficking for prostitution and egg donation for research are exploitive and thus should not be endorsed by feminists. Moreover, the failure to name such practices as exploitive serves to normalize and extend them, thus leading to the exploitation of more women. [5: 6]

There are cases of “egg donation for research” that are alarmingly similar to trafficking for prostitution, as we shall soon see, and Widdows’ concerns are therefore important. Placing trafficking for prostitution next to egg donation for research, however, as if both were uniform practices that are exploitive “*qua* practices,” mirrors a tendency in the embryo debate to place murder next to embryo destruction. When an obviously questionable practice is placed next to a practice that you want to debate, the obviously questionable practice easily becomes a model for what the debated practice inherently is like. Such a model can of course be illuminative, but it can also dominate the arguments that are meant to support the similarity between the compared practices. The difference between the practices must therefore also be noticed, as it is the reason why they are placed next to each other. The obviously questionable practice is needed to expose the not so obviously questionable practice. But the claimed similarity between the practices often remains unclear. The similarity then relies on a steady supply of philosophical arguments, which will be questioned, which will be defended, and the debate continues.^1^

Widdows begins her article by discussing trafficking for prostitution, and then she turns to egg donation for research. Here, a second not entirely obvious comparison seems to be made, in that egg donation for research is exemplified by egg donation for research fraud. The story of how a Korean researcher, Hwang Woo-Suk, obtained large numbers of human eggs by coercing young female team members to “donate” eggs to him, and by illegally buying human eggs for his research, serves in the article as a paradigmatic example of “the practice of egg donation for research.”

These not entirely obvious connections between practices give rise to a question. How can trafficking for prostitution, and research fraud, be used as illuminating comparisons when we discuss the intrinsic nature of “the practice of egg donation for research”? What can make these comparisons seem plausible?

My proposal is that the thick ethical concept of exploitation is influencing the investigation into what egg donation for research is. Since the practice of egg donation for research is argued to be exploitive, it seems close at hand to illuminate what egg donation *is* by connecting it to trafficking for prostitution, and to illegal and coerced egg “donation” for research fraud. The latter practices seem to show more clearly what egg donation for research *is*, even if at first it did not seem obvious.

Debaters who argue philosophically what embryo destruction or egg donation *are*, sometimes emphasize what they see as these practices’ morally problematic aspects by using words like “intrinsically” and “inherently.” Combining essentialist vocabulary with thick ethical concepts can, paradoxically, make it sound as if a debatable case such as egg donation for research, by being “inherently exploitive,” or “exploitive *qua* practice,” was even more exploitive than the obvious cases. Essentialist words can make such a strong impression on us that we fail to see that it is the other way around. We do not say that the assassination of John F. Kennedy was inherently murder; it was murder. We do not say that slavery is intrinsically exploitive; slavery is exploitive. By emphasizing indisputability, the use of essentialist words often inadvertently reveals that the cases are debatable.

The legitimacy of the comparisons that Widdows makes between different practices depends on whether she can argue convincingly that egg donation for research *is* exploitive as a practice. Why? Because “exploitation” is the conceptual thread that allegedly runs through the different practices and makes it legitimate to place them next to each other. However, the search for a definition of exploitation that can work in the argument already seems to be guided by the need to describe the compared practices as exploitative. Unsatisfied with the fact that Marx’s notion of exploitation does not seem to work for this purpose, Widdows writes:

It is important to retain the elements of power, coercion, and subordination in any definition of exploitation, so that we rightfully can include cases such as Hwang’s junior female researchers. A definition of exploitation that focuses only on disparity of remuneration misses the example out. Particularly for feminists, this caveat is crucial. [5: 20]

The aim with developing a thick ethical concept of exploitation, then, appears to be to enable us to identify exploitation *when we see egg donation for research*. A definition of exploitation is required that *describes* egg donation for research and *justifies* condemning it. The feature that in the end is deemed crucial seems to be gender subordination of women. Just as women’s choice in prostitution is exercised under systematic limitations, since men’s rights over their bodies are systematically privileged, “the need for women’s eggs in the stem cell technologies was simply taken for granted, as if medical research, too, enjoyed systematically privileged access to women’s bodies” [5: 20]. This is proposed to explain also why exploitation of women in the stem cell technologies never was debated academically. I agree, of course, that such an important concern must be addressed. Here I only examine the possibilities of such a discussion and point out certain dangers of thick ethical concepts.

Does an account of exploitation in terms of gender subordination legitimize the connections drawn between trafficking for prostitution, Hwang’s research fraud, and the practice of egg donation for research? It seems to do so, if we view Hwang’s research fraud as a plausible example of egg donation for research. But his exploitation of female team members is a plausible example of “the practice of egg donation for research” only if we already view egg donation for research as inherently exploitive, or exploitive *qua* practice.

It seems to me that we are caught in a vicious circle of thick ethical concepts and essentialist vocabulary. Without an overview of what egg donation for research is or can become – it varies across legislations and is still in the making – the thick ethical account of exploitation seems to inform us that egg donation for research *is* exploitative *qua* practice and that condemning it is the *right* thing to do.

Concerns about exploitation of women who donate to stem cell research must be addressed. I hope the above considerations indicate the need for a more “contemplative” approach to these concerns, one that is open to differences between practices and to possibilities of change. Otherwise, we are easily exposed to the dangers of thick ethical concepts, which seem to be able to determine what we do not know for sure but need to discuss openly. I doubt that we can say what the practice of egg donation for research is, in definite singular form. The discussion probably needs to start in that uncertainty.

## The Invisible Patient

Let me confess my own ignorance. When I hear of egg and embryo donation for research, I take for granted an IVF context and a strict regulation. I do not immediately think of research fraud. Neither do I think of an unregulated egg market in the US, or about poor women in different parts of the world who undergo hormonal treatment and surgery to offer their eggs at underprice, to get some much needed extra money. In other words, when I hear of egg donation for research, I admit that I tend to overlook some rather clear cases of exploitation, which are at the forefront of the articles just cited, and which, of course, need to be addressed.

Nevertheless, my ignorance of certain practices of donation is not completely out of touch with reality. It is in touch with *some* practices of donation, which are not as obviously exploitive as those of which I am more ignorant. And even if also regulated IVF practices could be exploitive, may we not be able to modify them to counteract the risks? By not talking about “*the* practice of egg donation for research,” and by not construing it conceptually as inherently exploitive, we can become more open to the possibility that some present or future practices could be ethically better examples of egg donation for research than Hwang’s research fraud. But are such practices of donation, more worthy of imitation, possible at all? I now turn to this question.

I repeat, we assume an IVF context and strict regulation and control. The woman undergoes hormonal treatment for the purpose of producing eggs to be artificially fertilized and reinplanted in her body, hopefully resulting in one or more longed-for children. In connection with the IVF treatment, the woman is asked if she is willing to donate surplus eggs, or embryos, to some specified embryonic stem cell study. She is informed also that research results may, at some point, be commercialized.

Even after pregnancy, surplus eggs and embryos can continue to be of immense importance to the woman. Not only because she may need them in the future, but also because they are such intimately significant parts of her body. They can become her children. This presents us with a puzzling problem. Why would anyone be willing to donate such sensitive “reproductive tissue” to researchers who wish to develop new stem cell technologies? Especially if the researchers state that they plan to develop medical products from the tissue and offer these products on a market? It can almost sound as if donors voluntarily agreed to be exploited.

To understand the possibility of a willingness among some women undergoing IVF treatment to donate such sensitive parts of their bodies to a research institution, or to “the stem cell technologies,” I believe we need to bring in a figure that so far has been invisible: the patient.

In the critical accounts of egg donation considered above, there is no mention of the fact that the new “stem cell technologies” are meant to function as treatments for future patients. If patients are mentioned, in passing, as in Waldby and Cooper [6: 5, 16], the potential benefits of regenerative medicine are described as “highly speculative” and as “fantasy,” as if patients were practically irrelevant to the field of stem cell research. Legally, the recipient of the donation is some research institution, of course, with its connections to industry and commercial activities. This conglomerate will potentially derive huge economic and other benefits from women’s donations, making the relationship between donor and recipient appear suspiciously unequal, even exploitive. Why would women want to give away reproductive tissue to support research institutions and entrepreneurs? Is it because, as women, they are expected to sacrifice their wellbeing for the wellbeing of others?

A plausible answer, I think, is that the more humanly intended beneficiary of the donation often is the hitherto invisible patient. Egg and embryo donation for research can make a puzzling impression if we leave the patient outside of our field of view. Of course, the donor may consider medical research important and worth supporting, even if it does not benefit any patient. However, we should not overlook the fact that medical research as a whole is related to the treatment of patients, and that even basic research and negative results are important in this broader context. That is why I want to broaden our field of vision, so that we can see the possibility that the legal recipient of the donation mediates gift relationships that extend further; a possibility which can make the donation look different than we first suspected – less asymmetric and puzzling. Of course, the “gift” is not always free for the patient, but it can at least become available to many patients, and even availability can be considered a gift (“gift” is not used here in opposition to “for a fee”; cf. how works of art can be considered as gifts to humanity even though museums charge and books have price tags). In other healthcare systems, the gift would be free, and that is enough for my purposes, which are about seeing *possibilities* when our way of thinking prevents us from seeing them.

## The Intermediating Function of Research and Industry

One could suspect that I bring in the patient only to speak to common normative expectations that women should sacrifice themselves for the needs of others, and that I thereby support the exploitation of women as analyzed by Widdows in terms of gender subordination. My problem, however, is more about our way of looking at donation for research, our difficulty of understanding it, if we do not view medical research in a wider perspective. Altruistic blood donation is easy to understand from a human point of view because the recipient is a needy fellow being, a patient. But how can we understand altruistic donation to a research institution?

Donation for medical research can seem puzzling in the absence of the patient. That is why I bring in the patient who disappeared in the moral concerns that egg donation for research might be exploitive. So, once again, I am not arguing that women undergoing IVF have a care duty to support stem cell research because it will benefit patients in need, or that a donation would be appropriate to reciprocate the gift of IVF treatment. I am only considering the broader context in which a free will to give seems less puzzling or suspicious. What intermediates such a gift relationship from donor to patient, when the direct recipient of the donation is a research institution?

Perhaps a simile explains how we, often without being aware of it, rely on intermediaries who, in their turn, depend on us. It is common knowledge that our digestive tract contains roughly one kilogram of bacteria, without which many of the nutritive substances in the food we eat could not become available to us and our bodies. When we swallow the food, these bacteria are the first eaters, and we have to wait patiently until they have eaten. Even if we know this to be a fact, we do not consciously think that we swallow food to allow microorganisms in our bellies to eat first. We eat for various reasons, but usually unaware of the intermediating function of bacteria.

I suggest that we can look at research and industry as intermediators of gifts from donors to patients. I hope I do not appear condescending if I propose that researchers and entrepreneurs are the societal bacteria that are needed to make the donation available to the patient’s body. We may dislike the idea that our stomachs are full of bacteria, or we may dislike technocrats and capitalists. Still, we rely on bacteria, technocrats, and capitalists. Considered in this wider perspective, who is exploiting whom?

I am proposing a broader way of looking at donation for research that can make it look less puzzling. The proposal is that when someone freely supports medical research by donating tissue, it may be due to some level of awareness of the intermediating function of medical research. (This does not exclude other possibilities, e.g., in systems where the donation gives the woman better conditions for IVF treatment [7].) The contribution to research will, in the end, hopefully be a contribution to patients. Few, however, are clearly aware of the fact that virtually every successful medical treatment that research contributed to developing was finalized and made available to patients by the pharmaceutical industry. There are so many layers of interdependency at work, when we consider donation for medical research in a larger context. Even generally disliked layers are needed and play at least partially beneficiary roles within the system as a whole. Research alone cannot intermediate altruistic gift relationships from donors to patients. There has to be an industry too, and a healthcare system, and much else. Moreover, just as the proper functioning of bacteria in our digestive tract needs regulation in the form of a diet that supports the right balance of beneficial bacteria, the system of intermediation from donor to patient needs to be regulated and supervised, so that the interdependent actors function harmoniously together. We do not want a system where quacks are free to sell dangerous and ineffective substances to people who are ill, or where stem cell researchers obtain human eggs in any way they see fit. We are surveying a whole society that allows donors to give to patients, if they want, by donating “for research.”

Egg donation for research turns out to be more difficult to isolate as a separate practice than we first thought. Donation depends on a vast system of interdependencies, comparable to what needs to happen in concert in our bodies when we think that we are simply eating. Our concepts do not reflect all of these dependencies, on which they rely for their daily use. This is true not only of “eating,” but also of “donating for research,” and of most concepts. They are simpler than the interdependent realities and relationships that underpin their ordinary use. Having these easily neglected interdependencies in clear view, it becomes surprisingly difficult to isolate *separate* actors; to see who *actually* eats first and who eats last; to see who *truly* is superior and who is subordinate; to see who *in fact* is benefitting and who the *real* benefactor is.

The fact that our concepts are simpler than the interdependencies which their daily communicative use presuppose is not a shortcoming. It creates problems only when we expect that the concepts reflect all the relevant facts and relationships. Egg donation for research is a good example. Linguistically and legally such donation is, of course, “donation for research,” donation to some research institution. This is not denied. If this conceptually highlighted relationship is seen as the whole of the donation, however, donation for research can look puzzling and even suspicious “as a practice.” We fail to see the possibility that donating to a research institution can be like handing over a parcel to the post-office clerk. The immediate recipient, the research institution, although conceptually highlighted, can drop off as relatively uninteresting for the donor. We can *see* this possibility, although the concept represents the research institution (or “the stem cell industries”) as the only recipient.

Having seen that the concept of “donation for research” does not reflect what can make the donation meaningful for the donor – the patient – moral concerns about risks of exploitation in the stem cell technologies transform accordingly. The donation is no longer seen as a transaction between obviously unequal parties, since it is possible for the donor to merely *use* the direct recipient to give to someone else. Perhaps without being fully aware of it, the donor uses not only research, but a whole system of mutually dependent actors and institutions, such as industry, healthcare, regulation, and governmental supervision. This system can therefore, unexpectedly, be seen as subordinate to the needs of donors who wish to give to patients. Or, this subordination is at least an *aspect* of the relationship, like the subordination of bacteria with regard to human eating. We can always see the opposite aspect as well, if we want to, since we are considering interdependencies.

Let us sum up, before we move on. In the accounts of egg donation discussed above, the donating woman seems obviously subordinate to the recipient, the research institution, with its connections to “the stem cell technologies.” In that conceptual framework, where the patient is unseen, risks of exploitation appear almost *a priori*. Another way of looking at donation, however, is to see research, in conjunction with a whole system of interdependent actors, as *intermediating* gift relationships from donors to patients. The fact that this intermediation engages a multi-billion dollar conglomerate raises reasonable concerns, of course. If these concerns are discussed openly, and the practice is regulated and works within proper bounds, however, there is a possibility that the intermediating system can be *made* as irrelevant to donors as bacteria in our stomachs are to diners. This possibility does not rule out risks of exploitation, but the risks no longer appear *a priori*, as in the narrower conceptual framework mentioned above. My hope is that by broadening our view to include the patient, we will be able to discuss relevant risks of exploitation while dealing with the intellectual dangers of the thick ethical concept of exploitation.

## Risks of Exploitation when the Humanly Intended Recipient is the Patient

As I mentioned in the introduction, instead of developing a conceptual analysis of exploitation, as in Zwolinski and Wertheimer [8], this paper describes general intellectual dangers that the word “exploitation” shares with many other thick ethical concepts, especially when the conceptual framework within which we think does not embrace all the relevant features of the practice that we are discussing. Having seen how concerns about exploitation can sometimes be a product of our conceptual framework, which emphasizes the direct recipient of the donation, I now want to exemplify some concerns that a broader view of donation for medical research may raise. Given the self-reflective nature of what I call a “contemplative” approach, I will only suggest four hypothetical cases, and only as an exercise in seeing possibilities that can emerge when we are no longer dominated by a limited conceptual framework.

One risk of exploitation could be well-intentioned attempts to counteract intellectually projected risks of exploitation by paying women for their “reproductive services” to the stem cell technologies, or by giving them a share of future profits. That could establish a tight relationship with the wrong other party, at least if we look at the matter from the broader perspective proposed here. I do not claim that paying for services inevitably means exploitation, but such frameworks invite concerns. Payment accentuates the donating woman’s relationship to a relatively powerful other party, “the stem cell technologies,” which can make exploitation a constant issue. Moreover, since the patient is hidden in such frameworks, such an attempt to counteract risks of exploitation makes practice of the limited conceptual framework that probably projects the concerns from the beginning. However, if the transaction is with the IVF clinic, some women may view donation rationally to be in their own interest, as in an empirical study by Haimes et al. [7: 1211]: “For the interviewees, exchanging eggs for more treatment and therefore for a greater chance of having a baby is a reasonable thing to do.” There are many possibilities.

Another risk of exploitation has to do with the gender differences that Dickenson and Widdows mention. To support an altruistic will to give, a patient perspective may be emphasized. Given normative expectations on women to devote themselves to the needs of others, such a perspective can be a delicate matter to handle. Caution is required to avoid exaggerating patient needs to such an extent that not donating appears unfeeling. Another related risk is presenting the donation as a gift in return for IVF treatment. If an individual freely donates in gratefulness for IVF treatment, this may be alright in the individual case. Framing egg or embryo donation in terms of reciprocation, however, can make the donation seem expected rather than free. Given the normative expectations mentioned above, both an overemphasized patient perspective and a perspective of reciprocation could coerce donation.

After these two possible concerns about exploitation – economization and “sentimentalization” of donation – I want to mention two concerns that donors themselves might have. The first has to do with the fact there are forms of egg donation worldwide that clearly do seem exploitive. Women who donate eggs or embryos in the course of undergoing IVF treatment may worry that their donation goes to institutions that exploit women in other circumstances, perhaps in other parts of the world. Could their free donation support exploitation of less fortunate women? However, these concerns, if addressed openly, could put pressure on research and industry to take a more global responsibility for what could one day, perhaps, deserve to be called, in definite singular form, “the practice of egg donation for research.”

Another possible concern that donors may have is the following. If women (or couples) donate with the patient in mind, they can worry that research and industry will fail to honor the altruistic spirit in which they gave to research. Some actors in the system that makes the donation available to patients may prioritize interests that interfere with the intermediating role that the donor more or less consciously expected. Stem cell treatments will in many cases not be made available to the most needing patients’ bodies, for example, because companies do not believe it is in their shareholders’ economic interest. Let me repeat here what I said earlier, that even accessibility can be considered a gift, that treatment in some healthcare systems is free, and that an open discussion of concerns can change practices. All I want to do here is help us see possibilities when a dominating conceptual framework prevents us from seeing them.

This brief exercise in seeing possible moral concerns can appear inconclusive for regulatory discussions about egg and embryo donation for stem cell research. My aims here, however, are preparatory. I want to counteract a second polarized stem cell debate and to demonstrate a more self-reflective and “contemplative” approach to concerns about exploitation, where we examine also possible intellectual dangers in our own concepts of donation and exploitation. Achieving these aims meant surveying relationships that are presupposed rather than expressed by the concept of “donation for research.” I would like to support regulatory discussions about donation for research that can navigate the conceptual dangers that so easily polarize debates. I would also like to suggest that such broader discussions could consider protecting gift relationships that extend beyond research, through commercialization, all the way to future patients and to future healthcare opportunities.

## Protecting Human gift Relationships

This concluding section indicates human functions that altruistic donation for research can have, and which regulators could view as important to support. In a paper entitled, “Gifts of the Body and the Needs of Strangers,” Thomas H. Murray argues that “impersonal gifts acknowledge an entire realm of moral relationships and moral obligations wider than intimate, family ones, and wider still than legal, contractual ones” [9: 35]. If Murray is right and impersonal gifts acknowledge larger dimensions of life, then regulatory discussions about donation for stem cell research could benefit from not putting all the emphasis on individual rights and interests. Individual rights and interests are very important if the sole intended receiver of the donation is the comparatively powerful direct recipient. If donation for research is made with future patients in mind, however, regulation could aim also towards maintaining some buffering distance between donors and direct recipients. Regulation could strive to ensure that intermediators function so harmoniously together that donors need not worry too much about them, but can confidently donate with the patient in mind. This could be an overall aim of regulation: to support a free will to give to unknown others by protecting gift relationships that extend from donors to future patients.

Murray’s paper focused on blood donation where it is relatively easy to see patients as the recipients of the donation. Egg and embryo donation for stem cell research is more complex, partly because the donation is literally “for research,” and partly because so many conjoined scientific, industrial, governmental, and other intermediating efforts are required to make the donation available to the bodies of future patients. If we do not consider the intermediating function of research and industry, and how the literal features of the concept of “donation for research” can obstruct seeing this function, we could be tempted to conclude that

…the claims of the gift relation are destabilized by the fact that donors to stem cell research give not to a fellow citizen [as in blood donation] but to an increasingly capitalized life science sector, which depends more and more transparently on the generally unremunerated labour of the donor. [6: 13]

To avoid that this conceptually tempting view of “donation for research” becomes true, a possibility emerging from the broader outlook of this paper is that regulation could deliberately aim towards protecting donation for research that has patients in mind. Such regulation could enable the complexity of altruistic donation “for research” to illuminate, rather than obscure, how interdependent we are as donors, researchers, funders, industrialists, regulators, authority representatives, healthcare professionals, and patients. It could help us see what our concepts presuppose rather than express literally. I am not thinking of slogans such as, “Together we create better futures for diabetes patients,” which would overemphasize the patient perspective and could act as a form of coercion, as we saw above. I am thinking of well-regulated practices of donation as opportunities for people to cultivate large-mindedness and to acknowledge unselfishness as a human possibility. This implies that several parts of the regulation need to be considered together: not only those parts that deal specifically with donation for research, but also parts dealing with patentability, with biobanks in academic research and industry, with biomedical products, and much else.

In conclusion, it is noteworthy that “literal” views on egg donation “for research” tend to construe relationships in such a manner that donors appear to be the passive party while the recipients are the active ones. Instead of creating such passive donors vis-à-vis powerful recipients, regulation could support active donors to safely exercise altruistic donation with future patients in mind, through a well-regulated intermediary system. By seeing human gift relationships as streams moving through the intermediary system, transporting biotechnologically modified tissue from human to human, donation for research can “remind us that wealth is merely a means to an end, and that not all valuable things can be purchased”[9: 35]. I am not suggesting, of course, that regulation should define patients rather than research institutions as the legal recipients of the donations. However, regulatory discussions can be sensitive to perspectives that are larger than the regulation itself, and this can leave imprints on the regulation. We are envisioning a whole society that allows donors to give to patients, if they want to, by “donating for research.”

## Footnotes

1 Widdows’ use of the example of trafficking for prostitution is more complex, in that it is a rather obvious case of exploitation that nonetheless has been questioned by feminists who fear to appear paternalistic (another thick ethical concept). Widdows’ view is that these feminists fail to identify exploitation when they see it. What is compared, then, are two cases of (allegedly, equally clearly) failing to condemn exploitation of women.

## References

[1] Dickenson DL (2006). The lady vanishes: what’s missing from the stem cell debate. Bioethical Inquiry.

[2] Holm S (2015). Biobanking human embryonic stem cell lines: policy, ethics and efficiency. Monash Bioethical Review.

[3] Williams B (1985). Ethics and the limits of philosophy.

[4] Segerdahl, P. (2022). Debating embryonic stem cell research: handling moral concerns more gently. In Ethical inquiries after Wittgenstein, eds. Ondrej Beran, Salla Aldrin Salskov and Nora Hämäläinen. Springer, pages 173-188. 10.1007/978-3-030-98084-9_11.

[5] Widdows H (2009). Border disputes across bodies: exploitation in trafficking for prostitution and egg sale for stem cell research. International Journal of Feminist Approaches to Bioethics.

[6] Waldby C, Cooper M (2010). From reproductive work to regenerative labour: the female body and the stem cell industries. Feminist Theory.

[7] Haimes E, Taylor K, Turkmendag I (2012). Eggs, ethics and exploitation? Investigating women’s experiences of an egg sharing scheme. Sociology of Health & Illness.

[8] Zwolinski, M., & Wertheimer, A. (2017). Exploitation. In E. N. Zalta (Ed), *The Stanford encyclopedia of philosophy* (summer 2017 edition). Retrieved September 11, 2019, from https://plato.stanford.edu/archives/sum2017/entries/exploitation/

[9] Murray TH (1987). Gifts of the body and the needs of strangers. The Hastings Center Report.

